# Emergence and Applications of Base Metals (Fe, Co, and Ni) in Hydroboration and Hydrosilylation

**DOI:** 10.3390/molecules24173194

**Published:** 2019-09-03

**Authors:** Sem Raj Tamang, Michael Findlater

**Affiliations:** Memorial Circle & Boston, Department of Chemistry & Biochemistry, Texas Tech University, Lubbock, TX 79409, USA

**Keywords:** sustainability, catalysis, hydroboration, hydrosilylation, earth-abundant

## Abstract

Base metal catalysis offers an alternative to reactions, which were once dominated by precious metals in hydrofunctionalization reactions. This review article details the development of some base metals (Fe, Co, and Ni) in the hydroboration and hydrosilylation reactions concomitant with a brief overview of recent advances in the field. Applications of both commercially available metal salts and well-defined metal complexes in catalysis and opportunities to further advance the field is discussed as well.

## 1. Introduction

Catalysis employing transition metal elements is a powerful methodology that is now widely and routinely used in both academia and industry in the transformation of organic molecules into value-added materials [1,2,3]. The advantages of any catalyzed reactions over an uncatalyzed one typically include a measure of control over regio-, chemo-, and stereoselectivity. One indication of the prominence of transition metal catalysis in both academia and industry has been the recognition in the form of Nobel prizes awarded to celebrate discoveries that have facilitated major changes in the field of chemistry [4,5,6,7,8,9,10,11]. 

Precious metals, such as Platinum (Pt), Palladium (Pd), Rhodium (Rh), Ruthenium (Ru), and Iridium (Ir), have been catalytic workhorses for decades and are often at the frontiers of new achievements and breakthroughs in catalysis. These metals can accommodate the 2e^−^ redox processes, which are typically favored during organometallic catalysis. Examples of precious metals in the transformation of organic molecules include reactions, such as hydroboration [12], hydrosilylation [13,14], arylation [15,16], amination [17,18] etc. However, one major drawback associated with employing precious metals in industrial applications would be the high cost and limited availability of these metals. For instance, the current rates of a kilogram (Kg) of precious metals are $32875 (Pt), $52735 (Pd), $172843 (Rh), $52206 (Ir), and $8819 (Ru) [19,20]. On the other hand, the current price of a kilogram (Kg) of base metals are $29 (Co), Ni ($16), and iron ore ($0.09) [20]. 

Base metal catalysis has seen an explosion of interest in recent times [21,22]. Notably, significant progress has been made in application of base metals in reactions that were once limited to precious metals: arylation [23], amination [24,25], hydrosilylation [26,27], borylation [28] and hydrogenation [29,30,31]. Furthermore, main group elements [32] and organocatalysts [33,34] have also emerged as “greener” alternatives for similar applications. The growth in all these strategies can be attributed to the (often) low toxicity, relatively lower costs of operation, and inherent sustainability associated with such an approach [3]. However, first-row transition metals are prone to single electron transfer process, which can often lead to non-productive side reactions, limiting catalytic efficiency. Several approaches have been explored to help circumvent this shortcoming. For example, metal-ligand cooperativity has played a significant role in enabling first-row transition metal catalysis of reactions that undergo two-electron processes. There now exists a readily accessible library of complexes composed of combinations of first-row transition metals with various types of ligands that have effectively and efficiently catalyzed reactions that were once limited to precious metals [35,36].

This review aimed to update the readership on applications of base metals (iron, cobalt, and nickel) in selected hydroboration and hydrosilylation reactions. Significant progress has already been made in the field, and the importance of metal selection and ligand designs have been extensively highlighted and reviewed in detail within the past 5 years. For example, a recent review by Beller and co-workers highlights the use of cobalt-based pincer complexes as catalysts in hydroboration, hydrosilylation, transfer hydrogenation, and de(hydrogenation) [37]. Similarly, a review by Hu and co-workers focuses on the application of iron-based pincer complexes in a variety of catalytic transformations [38]; Darcel and co-workers review gives a rather comprehensive account of iron in hydrometallation reactions [39]. Chirik and co-workers have also recently reviewed applications of earth-abundant transition metals in the hydroboration and hydrosilylation of alkenes [40]. Therefore, this review article has not sought to provide a comprehensive review of base metals in catalysis but rather has focused on more recent developments of base metals (iron, cobalt, and nickel) in selected reactions, which have an opportunity to advance the field of base metal catalysis further. In particular, we have discussed selected topics in this field, with special emphasis on our contributions. After summarizing the field, we have presented a perspective on key features of existing catalytic systems and our ideas on how best to advance the field in the future. 

Sem Raj Tamang was born and raised in Singapore where he was introduced to chemistry in secondary school. He earned his B.Sc. in Chemistry and Mathematics in 2011 from Elmira College, NY. He then joined Dr. James D. Hoefelmeyer’s group at the University of South Dakota to study bond activation using Frustrated Lewis Pairs. In 2016, he joined Dr. Michael Findlater’s group at Texas Tech University to study earth-abundant transition metal catalysis. His current work includes the use of iron, cobalt, and nickel in hydroboration and hydrosilylation reactions. 

Michael Findlater was born in Bellshill, Scotland and raised in Bargeddie, a small village just outside Glasgow. He received his undergraduate degree (B.Sc. Hons. in Applied Chemistry) from the University of Strathclyde in 2003 and moved to the United States to pursue his doctoral studies. In 2008, Michael received his Ph.D. from the University of Texas at Austin where he had undertaken research in main group synthesis with Professor Alan H. Cowley. His postdoctoral work was carried out with Professor Maurice Brookhart at The University of North Carolina in Chapel Hill. In 2011, he began his independent research career at Texas Tech University working, broadly speaking, in the field of catalysis. He received tenure with promotion to Associate Professor in 2017.

## 2. Hydroboration 

Organoboranes are excellent reaction surrogates as they provide an important reaction intermediate that could be transformed into a myriad of value-added products [1,12,40]. The organoborane surrogates that result from hydroboration can be used further for coupling reactions (i.e., formation of new C-C bond or new C-X (heteroatom) bond), which are routinely applied in pharmaceutical development [41,42]. While it can often be the case that hydroboration reactions can occur without the aid of a catalyst, these reactions are often limited to borylating reagents, such as the highly reactive diborane (B_2_H_6_) [43] 9-borabicyclo [3.3.1] nonane (9-BBN) [44,45], borane-tetrahydrofuran (BH_3_⸳THF) [44,45] and borane dimethylsulfide (BH_3_⸳S(Me_2_) [44,46]. It should also be noted that both the borylating reagent and borylated product formed requires careful handling as they are sensitive to air and moisture. On the other hand, dialkoxyboranes, HB(OR)_2_, such as HBCat and HBpin, are much easier to handle due to their relative stability, and the desired products are generally air-stable. Most importantly, catalyzed reactions give a measure of control over regioselectivity, enantioselectivity, and chemoselectivity [46,47,48]. Our efforts in hydroboration chemistry employing base metals (Fe, Co, and Ni) have explored the reduction of carbonyls [49], Markovnikov selective functionalization of alkenes [50] and regioselective dearomatization of *N*-heteroarenes [51]. 

## 3. Iron-Catalyzed Hydroboration of Carbonyls

**Scheme 1:**
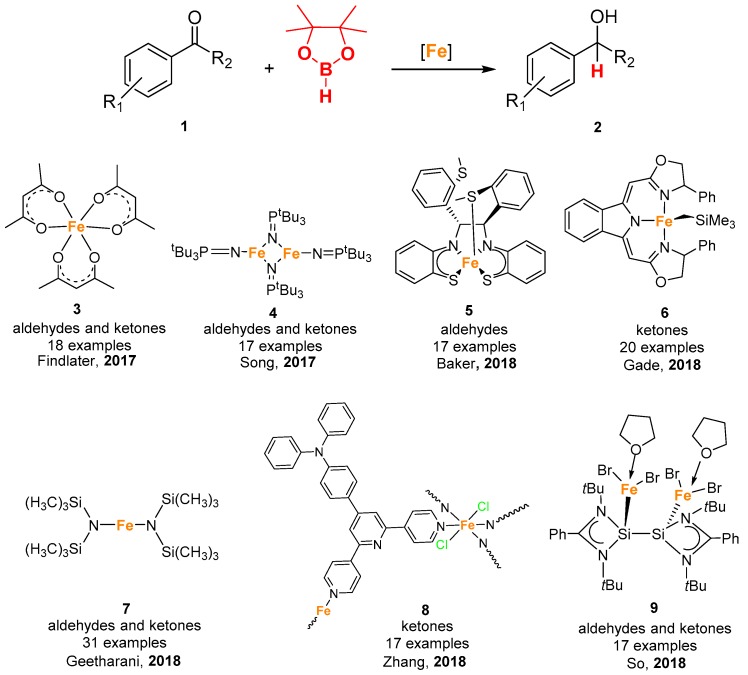
Examples of iron catalysts in hydroboration of carbonyls.

In 2017, we reported the use of an iron-based precatalyst for application in hydroboration of aldehydes and ketones [49]. We employed a simple activation strategy of commercially available Fe(acac)_3_ (**3**) with an equimolar amount of NaHBEt_3_ to generate a putative *“Fe-H”* species, which was found to be effective in accessing a viable catalyst for the room temperature hydroboration of aldehydes and ketones. At around the same time, Song and co-workers reported a homoleptic iron (II) bis(phosphoranimide) complex (**4**) catalyzed hydroboration of aldehydes and ketones as well [52]. Following these initial reports, many other studies employing iron-based catalysts for similar transformations have now subsequently appeared in the literature (Scheme 1). Baker and co-workers reported an imine coupled [Fe-N_2_S_2_]_2_ complex (**5**), which was found to be highly efficient in hydroboration of aldehydes at a catalyst loading of 0.1 mol% [53]. This catalyst was found to preferentially promote hydroboration of aldehydes over ketones; it was also found to be selective for the aldehyde moiety in intramolecular hydroboration of 4-acetylbenzaldehyde. While this obvious observation could be attributed due to the lack of reactivity of the catalyst (**5**) towards ketones, it could also imply the potentially important role of the ligand in showing such selectivity. Gade and co-workers reported a highly active chiral iron pincer complex (**6**), which catalyzed the reduction of α-haloketones with high enantioselectivity [54]. In particular, turn over frequencies (TOF) of > 4000 h^−1^ and tolerance of a range of functional groups were two very nice advances associated with this catalyst. Geetharani and co-workers [55] reported an iron (II), Fe[N{SiMe_3_}_2_]_2_ (**7**) catalyzed hydroboration of aldehydes and ketones. At the same time, Zhang and co-workers reported an iron (II) coordination polymer supported by divergent 4,2; 6′,4″ terpyridine (tpy) ligand (**8**); this heterogeneous catalytic system was used in the hydroboration of ketones [56]. So co-workers reported a silicon (I)- iron (II) dimer (**9**), which was formed after the treatment of an amidinatosilicon (I) dimer with FeBr_2_ in THF. This complex was active in the reduction of both aldehydes and ketones [57] (Scheme 1). The first report of iron nanoparticles (Fe_2_O_3_) catalyzed hydroboration of aldehydes and ketones was disclosed by Bose, Geetharani, and co-workers [58].

Analysis of the substrate scope revealed that all the catalytic systems were generally tolerant toward substrates bearing a wide range of functional groups: electron-withdrawing, electron-donating, and reducible functional groups. Aliphatic substrates were quantitatively reduced as well. Furthermore, substrates containing heteroatoms, such as X=O, and S, were also efficiently reduced to their respective alcohols. The reduction of nitrogen-containing substrates, such as 3-pyridinecarboxyaldehyde, was efficient (>99%) but 3-acetylpyridine showed little to no reactivity (<5%); substrates bearing -NMe_2_ showed poor to average reactivity. Notably, the α,β unsaturated bond of trans-cinnamaldehyde remained unchanged, and only one reduced product was observed except for our catalytic system, where we observed α,β-unsaturated 3- phenyl-2-propene-1-ol (major product) and 3 phenylpropan-1-ol (minor product) as a mixture (5.1:1). 

**Figure 1:**
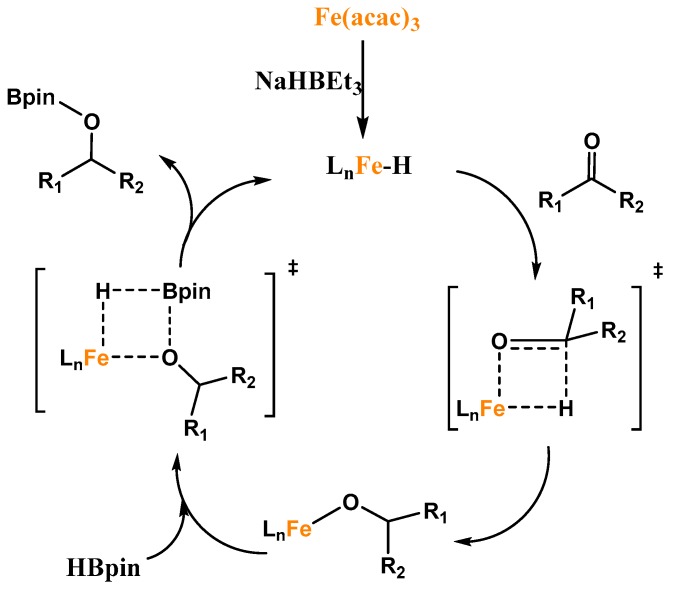
Proposed mechanism for hydroboration of carbonyls [27].

From a mechanistic perspective, we proposed that the transformation is speculated to proceed via the formation of a sigma bond metathesis, which is the generally accepted mechanism in hydrosilylation reactions (Figure 1). Our efforts in mechanistic studies by means of FTIR showed the formation of a new peak at 1701 cm^−1^, which was proposed to be the formation of a Fe-H bond upon addition of NaHBEt_3_ to (**3**); furthermore, stoichiometric addition of HBpin to the in-situ activated catalysts showed complete disappearance of the *ν*B-H band of HBpin (2580 cm^−1^) [49]. Similarly, Geetharani and co-workers observed a peak at 1658 cm^−1^ upon stoichiometric addition of (**7**) and HBPin; substituting HBPin to DBPin showed a shift in the peak to 1204 cm^−1^, suggesting the formation of a Fe-D species [55]. A detailed kinetic study by Baker and co-workers further revealed that the role of HBPin could be in the activation of the iron pre-catalyst (**5**) as the rate of consumption of HBPin was greater than that of aldehydes [53]. All these studies insinuate the generation of Fe-H intermediate species, and this might be the key step in the hydroboration of aldehydes and ketones. Alternatively, the transformation has also been proposed to proceed via the formation of a zwitterionic intermediate [57] without the need for the generation of a Fe-H intermediate. Therefore, a proper mechanistic study would help elucidate and would be a meaningful contribution to this growing field of study. 

The potential of iron in hydroboration catalysis has also been further demonstrated by targeting other functional groups aside from carbonyls; various functional groups, such as regioselective Markovnikov [59,60] and anti-Markovnikov [61,62,63,64,65,66,67] hydroboration of alkenes, regioselective 1,2 dearomatization of *N*-heteroarenes (*vide infra*) [68], regioselective hydroboration of alkynes [69,70,71] have also been achieved using iron catalysis. 

## 4. Cobalt-Catalyzed Hydroboration of Alkenes and CO_2_

Cobalt complexes have been also applied successfully in many organic transformation reactions [37,72,73,74,75]. There now exists many cobalt systems that have been used effectively as catalysts in hydroboration of organic substrates, such as alkenes [37,74,76], alkynes [77,78], carbonyls [74,79,80], imines [80], nitriles [76,77]. In particular, the regioselective hydroboration of alkenes is interesting as the formed alkyl boronate ester products could be further functionalized to value-added products that are useful in the synthesis of fine chemicals. At present, there already exist numerous cobalt systems that readily affords anti-Markovnikov borylated products [76,78,81,82,83,84,85,86,87]; the lack of Markovnikov selective reports is attributed to the less stable transition state of the reaction and the challenge associated with developing a general methodology applicable for a broad range of substrates bearing various functional groups.

**Scheme 2:**
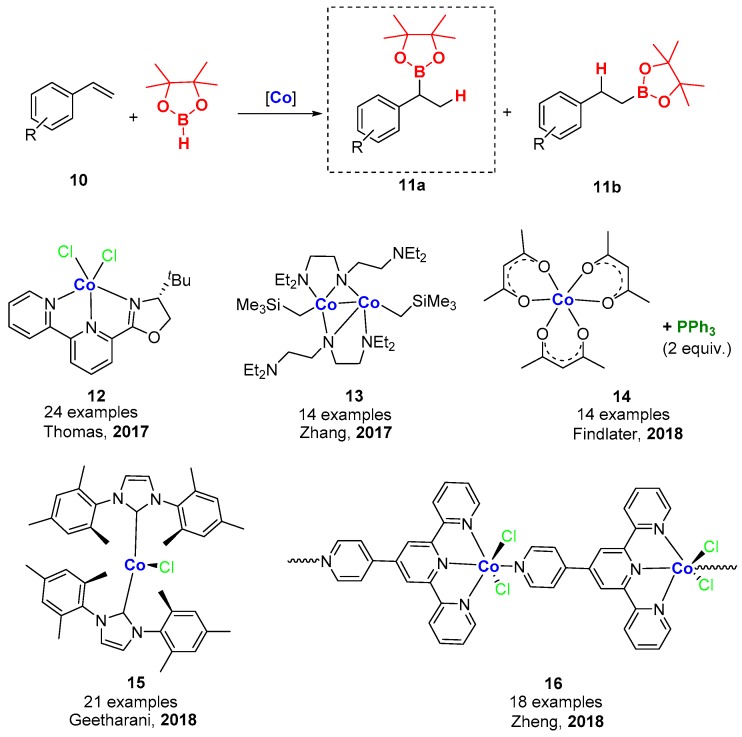
Examples of cobalt in Markovnikov selective hydroboration of alkenes.

Initial reports of cobalt in Markovnikov selective hydroboration of alkenes were made by Chirik (2015) [88] and Hollis (2016) [89]; however, only one example of Markovnikov addition was reported. In 2017, broad application of cobalt in Markovnikov selective chemistry was demonstrated by Thomas and co-workers (**12**, 24 examples with up to >98:2 regioselectivity) [90] and Zheng and co-workers (**13**, 14 examples with up to 98:2 regioselectivity) [84]. In 2018, we reported the use of cobalt in hydroboration of alkenes and additive-free hydroboration of carbonyls [50]. A simple and commercially available Co(acac)_3_ (**14**), precatalyst, in combination with NaO*^t^*Bu and PPh_3_ was found to be efficient for Markovnikov selective hydroboration of alkenes with up to 97:3 selectivity. Very recently, Co(IMes)_2_Cl (**15**) [91] and Co(II) polymer (**16**) [92] were also reported for Markovnikov selective hydroboration of alkenes; the former was also capable of catalyzing additive-free hydroboration of carbonyls. In particular, an impressive TOF of up to 47,520 h^−1^ was reported by Zheng and co-workers, and this made it the most efficient catalyst for hydroboration of alkenes to date [92] (Scheme 2). In all these examples, the ligand plays a very important role. For example, we observed that the addition of PPh_3_ as an ancillary ligand suppressed the formation of side products, such as ethylbenzene, and significantly increased the overall yield and regioselectivity for Markovnikov addition of styrene from 75:25 to as much as 94:6 [50]; Thomas [90] and Zheng [84] observed no reactivity in the absence of their NNN-based ligands. Furthermore, the addition of NaO*^t^*Bu or KO*^t^*Bu as an additive was an important component for catalysis as well; it was used to initiate catalysis presumably by activating the precatalyst and HBpin [93], though it should be noted that Geetharani’s *N*-heterocyclic carbene (NHC) supported Co(I) complex (**15**) [91] and Zheng’s NNN supported Co(II) complex (**13**) [84] could efficiently hydroborate alkenes under additive-free conditions. 

A survey of substrates showed that various electron-donating and electron-withdrawing groups were tolerated while maintaining regioselectivity for Markovnikov addition. Moreover, (**12**) and (**15**) were able to efficiently hydroborate substrates bearing -NMe_2_ and -NH_2_ functional groups [90,91]. On the other hand, regioselectivity was severely diminished for sterically encumbered substrates. For instance, hydroboration of 2,4,6-trimethystyrene with our catalytic system showed selectivity for anti-Markovnikov addition (20:80), while Geetharani observed a mixture of product in 46 (**b**):54(**l**) ratio with their catalytic system. Interestingly, (**15**) efficiently catalyzed the hydroboration of sterically encumbered α-methyl styrene with excellent regioselectivity for Markovnikov addition (92 (**b**):8(**l**)) in good yield (83%). Zheng’s Co(II) polymer (**16**) was also able to get regioselectivity for Markovnikov addition (75(**b**):25(**l**)), but the yield was only 26% [92]. In contrast, our catalytic system (**14**) gave 100% anti-Markovnikov regioselectivity with a yield of 39%. Reducible functional groups, such as esters and ketones, were tolerated as well. For instance, (**12**) and (**16**) showed excellent Markovnikov selectivity with no change in the ester moiety; yields up to 99% with the selectivity of up to 98(**b**):2(**l**) was reported. Similarly, both of these catalysts were also able to selectively hydroborate the alkene moiety when 5-hexen-2-one was subjected to their respective optimized hydroboration conditions; the ketone moiety did not get reduced during the reaction. While (**12**) afforded a 70 (**b**):30 (**l**) selectivity for Markovnikov addition, (**16**) showed a 10 (**b**):90 (**l**) selectivity for anti-Markovnikov addition. 

The reaction mechanism was probed using deuterium labeling experiments. Three independent experiments were set up with three different variables: (1) THF-*d*_8_, (2) Styrene-*d*_8_, (3) DBpin. The observations from these reactions implicated the possibility of alkene insertion into the cobalt hydride species followed by subsequent β-hydride elimination to generate the products; observations which were in agreement with Thomas and co-worker’s report. Geetharani and co-workers reasoned that since vinyl boronate ester was not observed as the side product, and the reaction favored Markovknikov selectivity, the insertion of alkene was into the Co-H bond rather than the C-B bond. Furthermore, comparison of the selectivity aliphatic and aryl alkene substrates revealed that the anti-Markovnikov selectivity for the aliphatic substrates could be due to the lack of π-benzylic interactions. 

Although Markovnikov selective addition can now be achieved for a broad range of aryl alkenes in the presence of a ligand and additive (or without additive), the challenge remains in obtaining Markovnikov selectivity for aliphatic substrates. Therefore, opportunities still exist to develop catalysts, which would show Markovnikov addition for both aryl and aliphatic alkenes. 

**Scheme 3:**
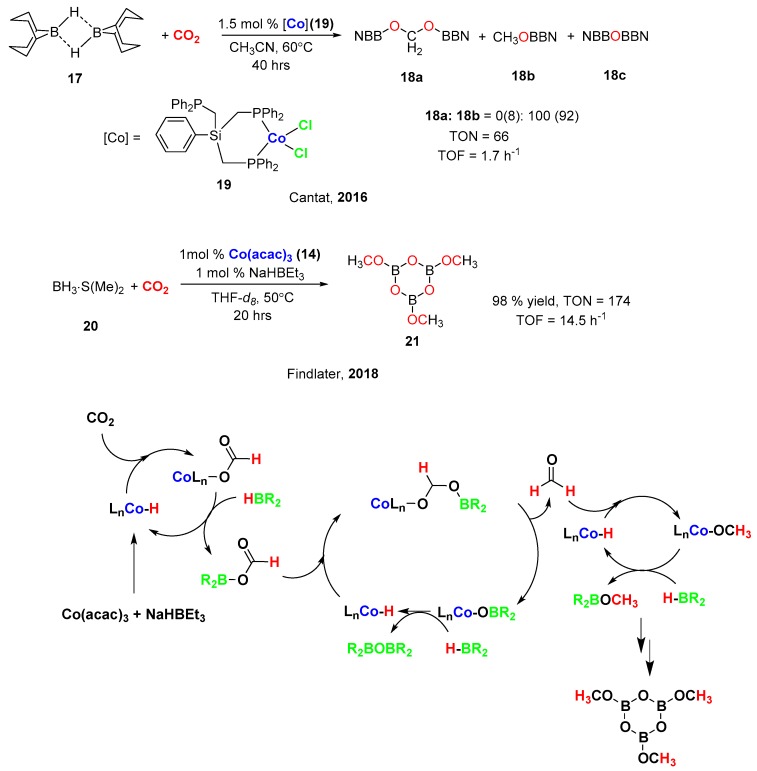
Examples of cobalt in hydroboration of CO_2_.

We also reported cobalt catalyzed reduction of CO_2_ via hydroboration (Scheme 3) [94]. Addition of a stoichiometric amount of NaHBEt_3_ to Co(acac)_3_ (**14**) generated a putative and catalytically active “Co-H” species, which reduced CO_2_ in the presence of various borylating reagents, such as HBPin, HBCat, and BH_3_⸳SMe_2_. We observed a 98% yield of methanol upon hydrolysis when BH_3_⸳SMe_2_ was employed as the reductant; turn over numbers (TON) of 174 and TOF of 14.5 h^−1^. Surprisingly, there is only one other report in which a cobalt catalyst was used for a similar transformation. Cantat and co-workers reported a reduction of CO_2_ with a combination of Co^2+^/ PhSi(YPPh_2_)_3_ (Y = CH_2_, O) complex (**19**) in the presence of 9-BBN. A conversion of 99% with predominant selectivity for methoxyborane was observed; TONs of 66 and TOF of 1.7 h^−1^ were reported; 100% selectivity for methoxyborane was observed at T = 40 h (92% selectivity at T = 24 h) [95]. Comparison of these catalysts with the most active examples in the literature suggests that there are ample opportunities to develop cobalt-based catalysts for the reduction of CO_2_ in the future and potentially further utilize CO_2_ as a C1 building block. 

The preliminary mechanistic study revealed that the transfer of hydride readily occurs as ^11^B NMR shows the peak for free BEt_3_ upon activation of the precatalyst. Moreover, we did not observe the formation of BH_4_^−^ species during catalysis [94]. Therefore, with reference to the literature, we proposed the initial formation of a cobalt formate complex after insertion of CO_2_ into the in-situ generated Co-H bond to generate formoxyborane, which inserts into the Co-H bond to form a metal acetal species, which can undergo β-alkoxy elimination to generate formaldehyde, which again undergoes insertion into the Co-H bond to give methoxyborane, which undergoes further transformation to give the final product, trimethoxyboroxine (Scheme 3). 

## 5. Nickel-Catalyzed Hydroboration of *N*-Heteroarenes

**Scheme 4:**
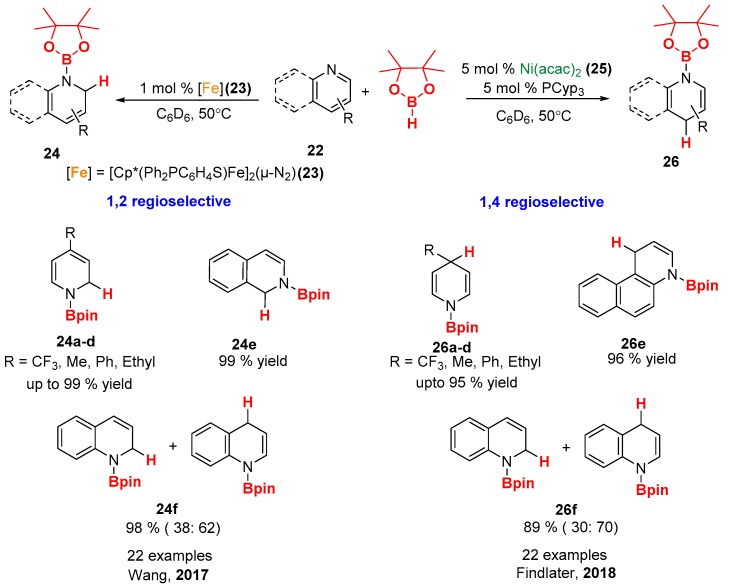
Comparison of [Fe] and [Ni] in regioselective hydroboration of *N*-heteroarenes.

**Figure 2:**
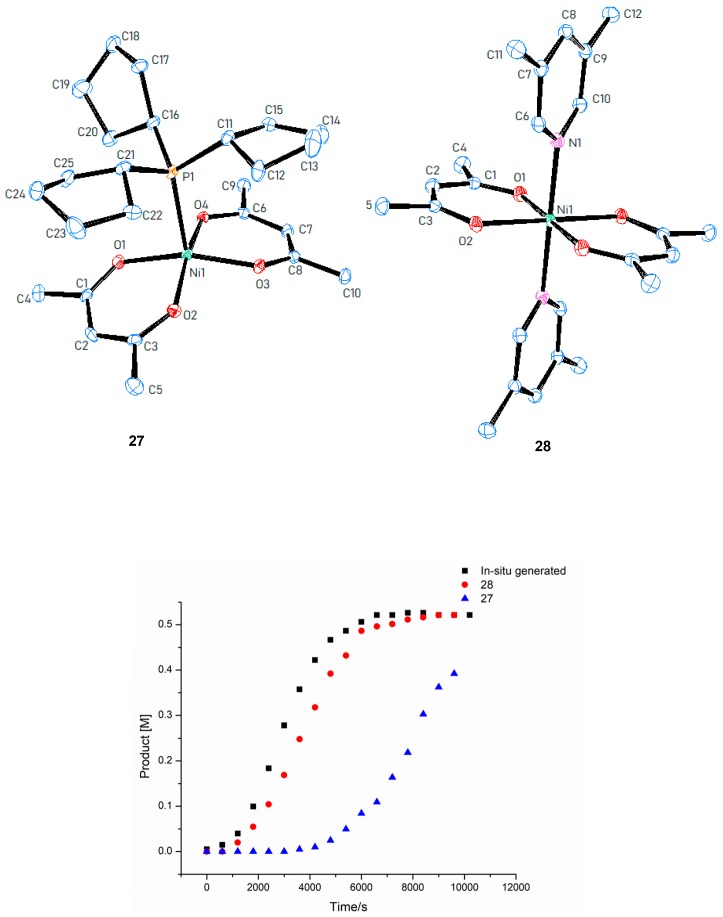
Top (left) solid-state structure of Ni(acac)_2_/ PCyp_3_ (**27**) complex; (right) solid-state structure of Ni(acac)_2_/lutidine complex (**28**); (Bottom) Kinetic profile of the reduction of lutidine in the presence of an in-situ generated catalyst, **27** and **28** [51].

Nickel can be considered as the ‘dark horse’ of first-row transition metals in catalysis and has found widespread use in organic chemistry [96,97]. Nickel catalysis in hydroboration has been reported for reduction of CO_2_ [98,99,100,101,102], carbonyls [103,104], nitriles [105] and alkenes [86,106,107,108,109]. In a related transformation, the dearomatization of *N*-heteroarenes to afford dihydropyridines (DHPs) is an important transformation as DHPs are the backbones of various pharmaceutically relevant molecules [110,111]. We recently disclosed the first example of nickel-catalyzed 1,4 regioselective dearomatization of *N*-heteroarenes [51]. This system complements the iron-catalyzed 1,2 regioselective dearomatization of *N*-heteroarenes reported by Wang and co-workers [68] (Scheme 4). A simple combination of Ni(acac)_2_ with tricyclopentylphoshine (PCyp_3_, 1 equiv.) resulted in the formation of a five-coordinate Ni complex (**27**). Similarly, a combination of Ni(acac)_2_ with pyridines resulted in the formation of octahedral bis(heteroarene) complexes (**28**) (Figure 2, Top).

The combination of Ni(acac)_2_/PCyp_3_ proved to be effective and efficient in catalyzing hydroboration of a wide variety of substrates while exhibiting relatively broad functional group tolerance. In particular, this was also the first system to demonstrate a general 1,4-hydroboration of *N*-heteroarenes for a number of para-substituted substrates (**26a**–**d**); the only other known example was reported by Hill and co-workers whereby they showed a single example in which a mixture of regioisomers was observed with the reaction favoring the formation of 1,2-dearomatized product (81:19) [112]. One major drawback for our catalytic system was the limited activity observed in the presence of halogens. We observed no reaction with 3-iodopyridine, and dehalogenation of 3-fluoropyridine was observed alongside with the 1,4-reduced product. Therefore, there still exists an opportunity to develop Ni-based catalysts, which could tolerate halogens and, at the same time, complement our study by catalyzing 1,2-hydroboration of *N*-heteroarenes. 

The role of the ligand is very important in this transformation as we observed little to no reduction of pyridine in the absence of it. Similarly, Wang and co-workers also showed the importance of ligand in their catalysis from a mechanistic perspective [68]. Mechanistic studies revealed that the phosphinothiolate ligand facilitated the hydride transfer via S->B interaction with concomitant capturing of the borenium ion after cleavage of the B-H bond, which was subsequently transferred to the reduced *N*-heteroarenes to generate the final product. Our preliminary mechanistic study revealed that the presumed precatalyst (a five-coordinate Ni(acac)_2_/ PCyp_3_ complex (**27**)) resulted in a rather sluggish catalytic reaction; the bis(lutidine) nickel complex (**28**) afforded a kinetic profile much more closely resembling that of the original in-situ catalytic reaction (Figure 2, bottom). The kinetics showed that the reaction was 1st order with respect to [Ni], 0th order with respect to *N*-heteroarene, and saturation kinetics for HBPin (1st order till 1.5 equivalence of HBpin, and 0th order after that). While these data provide valuable information, such as the binding of the *N*-heteroarene to the Ni center to be not rate limiting, we were still unable to fully elucidate the role of phosphine and, thus, we were unable to provide an alternative to the proposed 1,5-hydride shift by Gunanathan and co-workers [113]. More detailed mechanistic studies and computational analyses are currently underway in our group to better elucidate the reaction mechanism and most importantly identify the role of added phosphine.

## 6. Iron-Catalyzed Hydrosilylation 

Analogous to hydroboration, hydrosilylation reactions are also relied upon extensively in the industry. It provides direct access to organosilicon compounds, which have important roles in our everyday lives. Hydrosilylation of alkenes gives rise to alkyl silanes, which can be further transformed into value-added products, such as cosmetics, silicone rubbers, and textiles. Platinum-based catalysts, such as Speier’s catalyst [114], Karstedt’s catalyst [115] and modified Karstedt’s catalyst [116] are at the core of revolutionizing the silicon industry [117,118,119]. However, the cost associated with Pt (and other precious metals) due to its low abundance in nature and its toxicity has become an underlying issue for future sustainable development of such technology. Therefore, investigation of earth-abundant transition metals in the hydrosilylation of alkenes is of fundamental interest and has seen significant progress in recent times [26,38,39,120]. A recent review by Chirik and co-workers gives a very comprehensive report of applications of base metals in the hydrosilylation of alkenes [40]; many other reviews on this particular transformation also already exist. However, it should be noted that a comprehensive overview of hydrosilylation in the transformation of other organic substrates has not been reviewed as much or given much attention as compared to alkenes [121]. 

**Scheme 5:**
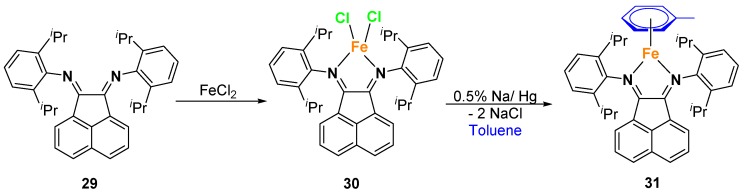
Synthesis of ^dpp^BIANFeCl_2_ (**30**) and ^dpp^BIANFe(η^6^- C_7_H_8_) (**31**) [68].

Our efforts in hydrosilylation chemistry with iron has explored hydrosilylation of aldehydes [122], ketones [122], esters [123] and imines [124]. To facilitate this chemistry, we have been studying metal-ligand cooperativity. In particular, we have been exploring the reactivity of iron complexes, which incorporate non-innocent redox-active ligands and pincer ligands respectively. We recently disclosed the syntheses of two iron complexes supported by a non-innocent redox-active ligand: ^dpp^BIANFeCl_2_ (**30**) and ^dpp^BIANFe(η^6^-C_7_H_8_) (**31**) [122] (^dpp^BIAN = 1,2-((bis-2,6-diisopropylphenyl)imino)acenaphthene) (Scheme 5). The fusion of the α-diimine fragment with the naphthalene ring is an important ligand design principle: (1) it serves as an electron reservoir during catalysis, thus supporting the metal center in various oxidation states during the catalytic cycle; (2) the rigid structure imparts a degree of stability to the complex.

^dpp^BIANFe(η^6^-toluene) (**31**) was found to be effective in the hydrosilylation of aldehydes and ketones under activator and solvent-free conditions at a loading of 1 mol%; broad functional group tolerance was observed. Single crystal data revealed that the 18-electron diamagnetic ^dpp^BIANFe(η^6^- C_7_H_8_) (**31**) exhibited changes in bond lengths (C-C = 1.400(4) Å and C-N = 1.343(3) Å) when compared to the 14-electron paramagnetic ^dpp^BIANFeCl_2_ (**30**) (C-C = 1.506(6) Å, C-N = 1.283(6) Å). Further characterization by ^57^Fe Mössbauer at 80 K revealed a doublet with an isomer shift (δ) of 0.45 mm s^−1^ and a quadrupole splitting (ΔEq) of 0.41 mm s^−^^1^. While these data provide valuable information for the proper assignment of oxidation states of the metal center, further studies are still required [125]. Hoyt and co-workers disclosed a more detailed study of ^dpp^BIANFe(η^6^-C_7_H_8_) (**31**) using a combination of X-ray diffraction, solid-state Mössbauer, solution-state magnetic calculations, and density functional theory (DFT) calculations [126]. The overall conclusion of these studies strongly implied that an oxidation state of +1 would be appropriate due to the transfer of an electron from the metal center to the BIAN ligand, thus forming (^dpp^BIAN^−^^1^)Fe^I^(η^6^-C_7_H_8_). They also demonstrated that ^dpp^BIANFeBr_2_ upon activation with LiCH_2_SiMe_3_ could be used effectively in the hydrosilylation of 1-hexene. 

**Scheme 6:**
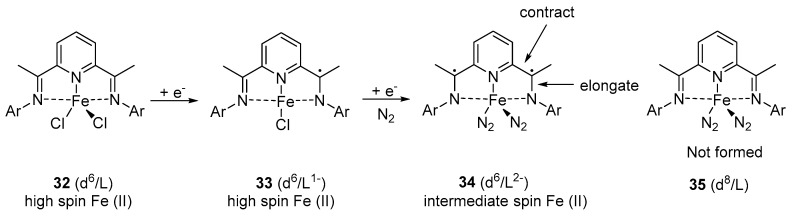
Understanding the concept of redox non-innocence [127,128].

**Scheme 7:**
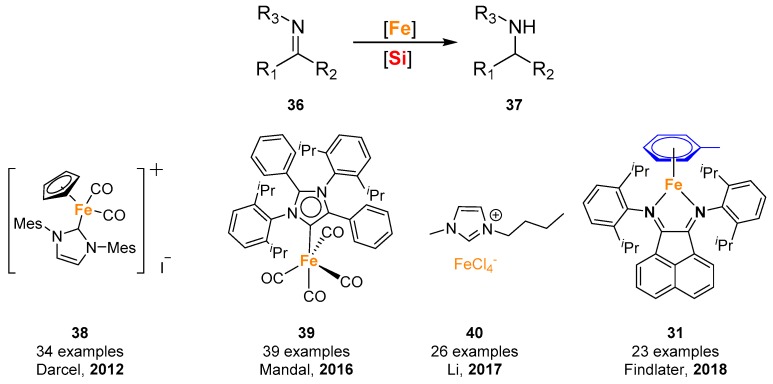
Examples of iron-based catalysts in the hydrosilylation of imines.

While the hydrosilylation of aldehydes and ketones has been extensively studied, there exist only a handful of examples of iron-catalyzed hydrosilylation of imines [129,130,131]. The reduction of imines to amines or formation of amines, in general, is an important transformation due to the potential of amines to be utilized as building blocks for organic molecules and biologically active molecules. Interestingly, it should be noted that none of the reported iron-catalyzed studies are based on redox-active ligands (see Scheme 6 for a pictorial depiction of redox non-innocence) but instead non-innocent ligands, i.e., ancillary carbene ligands (**38**, **39**) [129,130] and ionic liquids (**40**) [131] (Scheme 7). Darcel and co-workers reported an NHC carbine-based iron (II) catalyst (**38**) for visible light assisted hydrosilylation of both aldimines and ketimines under solvent-free conditions [129]. A wide variety of substrates bearing electron-donating, electron-withdrawing, heteroatoms, and sterics were tolerated. However, the reaction in the presence of R = NO_2_ (10% isolated) and -CN (15% isolated) gave limited reactivity. Mandal and co-workers reported an NHC carbine-based iron (0) catalyst (**39**) for the reduction of both aldimines and ketimines at room temperature with TON of up to 17,000 [130]. Substrates bearing a wide variety of functional groups, such as electron-donating, electron-withdrawing (inclusive of NO_2_ and CN), halogens, esters, and reducible functional groups, were well-tolerated. This methodology could further be extended to imines bearing sugar moieties; though the hydroxyl group had to be fully protected for efficient conversion. Moreover, chemoselective experiments showed that the reaction favored the reduction of the imine bond over reducible functional groups, such as aldehyde and ketones. Li and co-workers utilized an ionic liquid-based iron catalyst, 1-butyl-3-methylimidazolium tetrachloride iron (**40**), for the hydrosilylation of imines (aldimines) and reductive amination of aldehydes. The control experiments showed that the reaction in the absence of Fe yielded 37% of a secondary amine and 76% with just FeCl_3_; the combination of both 1-butyl-3-methylimidazolium and FeCl_3_ yielded 88% of secondary amine under the same reaction condition [131]. This was the first example of an imidazolium-based ionic liquid reduction of imines. This catalytic system efficiently reduced the hydrosilylation of imines under an air atmosphere. Though general tolerance towards substrates bearing various functional groups was observed, moderate activity was seen for substrates bearing -NO_2_ and -CN. We were also pleased to observe that ^dpp^BIANFe(η^6^-toluene) (**31**) could catalyze the reduction of aldimines into secondary amines at a loading of 1 mol% in the presence of triethoxysilane (Et_3_OSiH); no activator was needed to initiate catalysis [124]. Good to excellent activity was observed in the hydrosilylation of aldimines bearing various functional groups, while only moderate activity was obtained with a ketimine substrate (42% yield). 

Mechanistic studies proposed by Mandal showed that the reaction mechanism for the hydrosilylation of imine followed the typical Chalk-Harrod mechanism for hydrosilylation reactions [130]. A combination of theoretical and experimental evidence suggested that the oxidative addition of the silane occurred after one of the π acidic CO ligand left; this generated the active iron hydride species, which could be accessed by ^1^H-NMR. Subsequent hydride migration of the imine and reductive elimination generated the desired product.

**Scheme 8:**
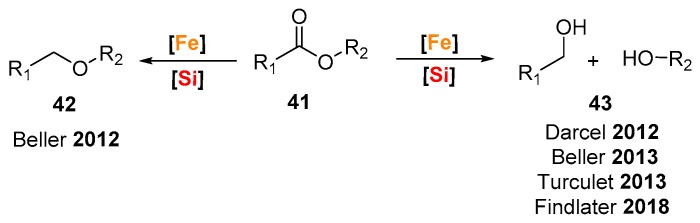
Examples of iron-catalyzed hydrosilylation of esters.

The generic reduction of carbonyls into alcohols has been studied extensively using base metals using hydrosilylation, hydroboration, and hydrogenation. This transformation is important as it has widespread use in the chemical industry [1]. The first example of a well-defined iron-catalyzed hydrosilylation of esters was reported in 2012 by Darcel and co-workers [132] (Scheme 8). A mixture of both esters and ethers were observed when [CpFe(CO)_2_(IMes)]^+^[I]^−^ was used initially as a catalyst in the presence of PhSiH_3_. Further optimization revealed that [CpFe(CO)_2_(PCy_3_)]^+^[BF_4_]^−^ in combination with PhSiH_3_ could be used to selectively reduce esters into their respective alcohols under visible light irradiation. In complementary work, Beller and co-workers were able to reduce esters into ethers, a very challenging transformation, using triiron dodecacarbonyl (Fe_3_(CO)_12_) and 1,1,3,3-tetramethyldisiloxane (TMDS) [133]. The methodology was extended to encompass a broad scope of substrates, which included cholesteryl nonanoate, which is used industrially in cosmetics applications. This finding was significant as it represents a catalytic alternative to the synthesis of ethers aside from the traditional Williamson ether synthesis, which has its limitations. Other examples that are capable of achieving similar transformation include Mn [134], Ti [135], Ru [136] and In [137]. Comparison of the structural component of these molecular complexes, except for In, showed that a common feature of these complexes featured either cyclopentadienyl (Cp) or CO as ligands. This could presumably be an important feature in achieving a reduction of esters into ethers. The follow up work by Beller and co-workers further investigated ligand effects on catalysis; screening of various N ligands with different Fe salts resulted in the optimal choice of Fe(Stearate)_2_ and 1,2-diaminoethane for the reduction of esters to alcohols [138]. Interestingly, Fe_3_(CO)_12_ in the presence of 1,2-diaminoethane resulted in no reactivity even though it is an active catalyst for reduction of esters to ether (*vide supra*). Turuculet and co-workers developed an impressive *N*-phosphinoamidinate iron (II) amido precatalyst, which was found to be active in the hydrosilylation of aldehydes, ketones, and esters [139]. It should be noted that catalysts, which are active in the reduction of aldehydes and ketones, are typically ineffective or have not been demonstrated to reduce esters.

General analysis of the substrate scope for the esters to alcohol reduction showed that electron-donating, electron-withdrawing, and steric were well tolerated. Substrates bearing heteroatoms X=S showed good conversion (>97%), while *N*-heteroatom substrates showed poor (35%) to excellent (>99%) conversion. Aliphatic esters were also reduced in an average to excellent conversion (51–88%), and cyclic esters were also reduced in moderate yield. Similarly, the selective reduction of esters into ethers could also be efficiently achieved in with various functional groups. The good activity was observed for aryl esters (70–85%) and aliphatic esters (60–73%), while moderate activity was observed for alicyclic esters (50%).

We were very surprised to find that there were no reports of ester reduction via hydrosilylation after 2013, though, alternative methodologies, such as hydrogenation, were reported [140,141,142,143,144,145,146]. When we subjected benzyl benzoate to catalytic reduction in the presence of polymethylhydrosiloxane (PMHS) and ^dpp^BIANFe(η^6^-toluene) (**31**), only 29% of benzyl alcohol was obtained [123]. Wangelin and co-workers reported that addition of *n*-BuLi (3 equiv.) to ^dpp^BIANFeCl_2_ generated an anionic [Fe] species, which was found to be an active hydrogenation catalyst for alkenes [147]. Inspired by this report, we applied the same conditions successfully to the hydrosilylation of esters and found that a broad scope of the substrate could be amenable to this transformation. Aryl esters bearing electron-donating, electron-withdrawing, and steric were well tolerated. Moreover, we observed excellent conversion with alicyclic and aliphatic esters as well; poor activity was observed with pyridyl substrates, such as ethyl nicotinate (7%) and ethyl-6-methylnicotinate (10%). This could be due to the pyridyl substrates acting as a ligand and coordinating with the iron center. 

**Scheme 9:**
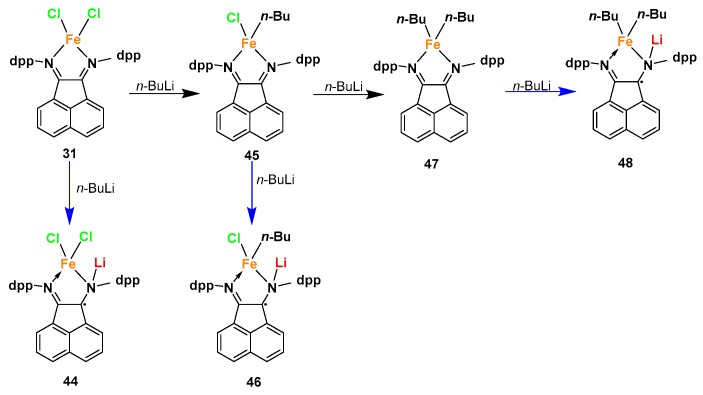
Proposed pathway at various addition of *n*-BuLi [122].

Our initial attempt to decipher the mechanism using electron paramagnetic resonance (EPR) revealed that rather than a 3-electron reduction to generate an anionic Fe species, the formation of a radical anion of the ligand might be taking place. Radical dearomatization of ^dpp^BIAN ligands in the presence of *t*-BuLi [148,149] and alkylation of ^dpp^BIAN with *n*-BuLi due to the formation of organic radicals have been reported in the literature [150,151]. Titration of *n*-BuLi to a solution of ^dpp^BIAN and ^dpp^BIANFeCl_2_ in toluene independently indicated a competition between ligand metathesis and complex reduction at each addition of *n*-BuLi (Scheme 9). The influence of the choice of ligand was also demonstrated as comparison of non-innocent redox-active ligands (^dpp^BIAN, PDI, ^mes^DAB) with redox innocent NHCs ligands (ipr and SiPr) revealed that the combination of FeCl_2_ with redox non-innocent ligands was more effective (yields up to 97%) than the redox innocent ligands [123]. Further work is undergoing in our group to isolate the reaction intermediate to complement our EPR findings and draw up conclusive mechanistic insights. 

## 7. Hydrosilylation Catalyzed by Iron- and Cobalt-Pincer Complexes

**Scheme 10:**
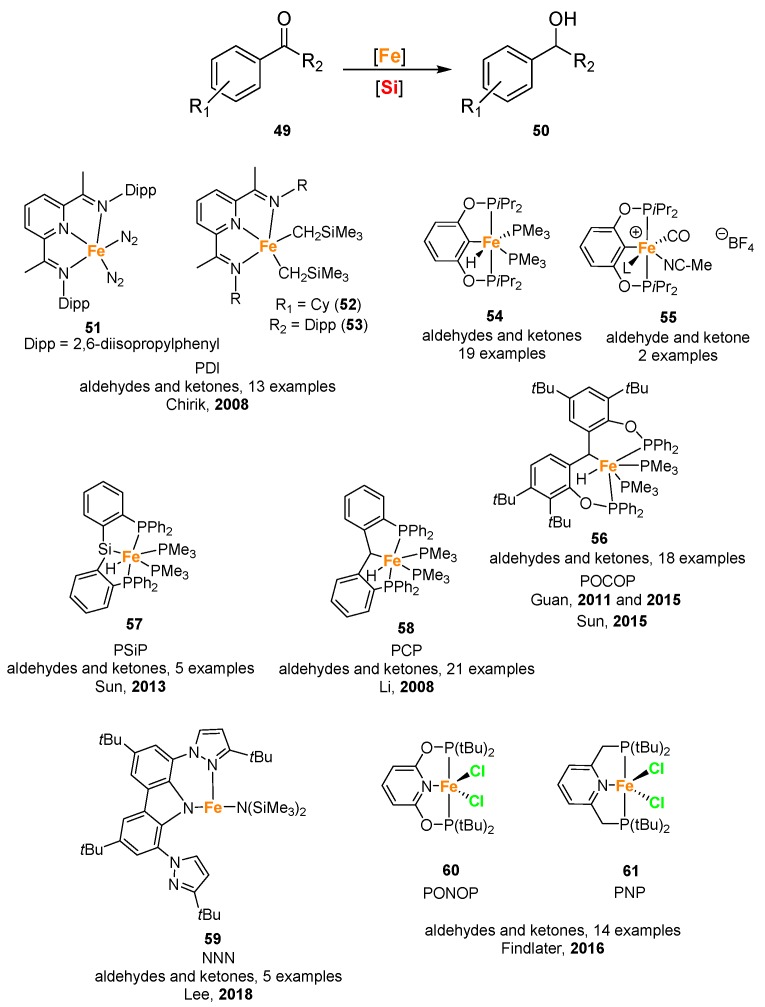
Examples of iron pincer catalysts in the hydrosilylation of aldehydes and ketones.

Our interest in the influence of ligands on catalysis includes pincer ligands as well. The ease in fine-tuning catalytic activities by modifying the ligand motifs makes this class of ligand very attractive. We have explored iron and cobalt complexes, with pincer ligands, for applications in the hydrosilylation of carbonyl moieties [152]. There are various types of pincer ligands that have been reported and studied in detail [153,154,155,156,157,158,159]. Some examples of iron pincer complexes in hydrosilylation have been reported by Chirik and co-workers in which the bis(imino)pyridine (PDI) iron dialkyl complexes (**51**–**53**) were employed for the hydrosilylation of aldehydes and ketones [160]. Comparison of (**52**) and (**53**) in the hydrosilylation of electron-donating and electron-withdrawing carbonyls (aldehydes and ketones) revealed that **52** was more active as the efficient conversion was observed with just 0.1 mol% loading of catalyst, while 1 mol% of **53** was needed for the same effectiveness. However, **53** was a better catalyst for aliphatic ketones. Guan and co-workers reported a POCOP-based iron-hydride complex (**54**) [161] and POCOP-based cationic iron complex (**55**) [162] for similar applications in the presence of (EtO)_3_SiH. Hydrosilylation of several aldehydes bearing various functional groups could be efficiently achieved with (**54**). However, the reduction of ketones varied according to the functional groups. For instance, electron neutral substrate could be reduced within 4.5 h, the electron-withdrawing group substrate could be reduced within 9 h, and the electron-donating group took 48 h. Moreover, no reaction was observed in the presence of sterics. (**55**) was sluggish in the hydrosilylation of aldehydes and ketones; both reactions took 24–48 h for quantitative conversion. The combination of PSiP (**57**) [163], PCP (**58**) [164] and POCOP (**56**) [165] iron hydride complexes were reported by Li and Sun, respectively; they were also found to effectively reduce aldehydes and ketones with (EtO)_3_SiH as the silylating reagent. Very recently, Lee and co-workers reported an NNN pincer ligand-based iron (II) complex (**59**) with TOF up to 11 min^−1^ (Scheme 10) [166]. 

**Scheme 11:**
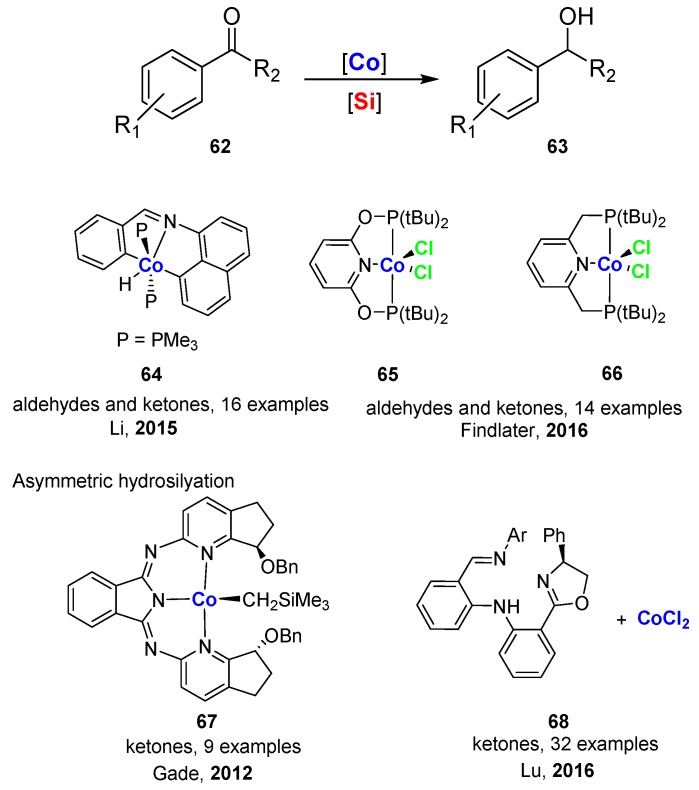
Examples of cobalt pincer catalysts in the hydrosilylation of aldehydes and ketones.

On the other hand, the applications of cobalt pincer complexes in the hydrosilylation of carbonyl moieties are underdeveloped compared to iron pincer complexes (Scheme 11). In 2012, Gade and co-workers reported pincer cobalt catalysis featuring a chiral 1,3-bis(2-pyridylimino)isoindolate ligand (**67**); high yields and enantioselectivity up to 91% ee was observed. However, it should be noted that steric-induced substrate was only moderately reduced with diminished ee (40%), and no reaction was observed when large groups were present in the ortho-positions [167]. Moreover, while the substitution of the R group at the backbone of the ligand did not influence the enantioselectivity, a drastic difference was seen when the R group at the pendant moiety was substituted. For instance, when the R group was changed from methyl to methoxymethyl acetal (MOM), the ee of 1-phenylethanol decreased drastically from 88% to 23% [167]. In 2015, Li and co-workers reported the synthesis of hydrido cobalt (III) complex (**64**), which was used in the hydrosilylation of aldehydes and ketones. The catalytic loading for the reduction was dependent on the substrates. For instance, aryl aldehydes and furfural could be reduced to their respective 1° alcohol readily with 1 mol% of catalyst; steric-induced electron-donating groups and ketones required 5 mol% of catalyst loading [168]. Lu and co-workers also reported asymmetric hydrosilylation of ketones in 2016. The combination of CoCl_2_ with chiral iminophenyl oxazolinylphenylamines (IPOPA) (**68**) showed excellent activity with high enantioselectivity of up to 99% ee. The substrate scope showed that the reaction was enantioselective even when steric-induced ketones (ortho-substituted) were reduced; however, more sterically hindered ketones, such as iso-butyrophenone and tert-butyl phenyl ketone did not yield any products. Heteroatoms, such as X=S and O, were also readily reduced with up to 93% ee, but the reduction of 2-acetylpyridine did not show any reactivity [169]. Therefore, there is an opportunity to develop cobalt catalyzed asymmetric hydrosilylation of acetylpyridine. Our work focused on utilizing ^tBu^PONOP and ^tBu^PNP ligand, which were conveniently synthesized via salt metathesis; metalation with anhydrous FeCl_2_ and CoCl_2_ readily afforded ^tBu^PONOP(MCl_2_) (**60**, **65**), ^tBu^PNP(MCl_2_) (**61**, **66**) complexes [152]. In-situ activation of the catalysts with NaHBEt_3_ (2 equiv.) generated a Co-H species, which was active in the hydrosilylation of aldehydes and ketones. No obvious trends were observed in determining which combination of ligand and MCl_2_ was the most effective. In general, (**61**) was a better catalyst than (**62**) for a variety of substrates. It showed moderate to good activity for aliphatic ketone, acetophenone, electron-donating, and electron-withdrawing aldehydes. 

We proposed a mechanism whereby the addition of NaHBEt_3_ was necessary to generate an active Co-H complex, which could activate the silane or the carbonyl moiety before hydrosilylation. Similarly, Lu and co-workers also proposed the necessity for the addition of NaHBEt_3_ to generate the active Co-H species [169] and Li’s hydrido cobalt pincer complex did not require the addition of NaHBEt_3_ or any additives [168]. Therefore, this could imply the need for a Co-H catalyst for effective and efficient catalysis. 

## 8. Outlook

Hydroboration and hydrosilylation represent just two examples amongst many types of hydrofunctionalization reactions that are actively being explored. Much of our work in hydroboration has focused on commercially available pre-catalysts, which eschew the norm of elaborate ligand design typical in the development of new catalyst systems. We aim to develop operationally convenient methods for facile catalytic applications. However, challenges remain in the use of commercially available metal salts, for example, how do we fine-tune regio- and chemo-selectivity without appropriate modular ligand support?

The growing interest in base metal catalysis is evident from the increase in reports available in the literature. This review article summarizes recent advances in hydroboration and hydrosilylation employing iron, cobalt, and nickel. While base metals are still far from replacing precious metals in everyday applications, significant progress has been made for their use in organometallic catalysis in an academic setting. However, more and more, it is becoming apparent that industrial use is becoming more attractive from both a cost and sustainability perspective [3]. 

Our interests in studying catalysis are not just limited to commercially available metal salts. We are also interested in understanding the fundamental underpinnings of metal-ligand cooperativity in catalysis. Our focus has been on the incorporation of redox non-innocent ligands (^dpp^BIAN) and pincer ligands (POCOP and PNP) into base metal complexes. These well-defined systems have been effectively applied in hydrosilylation reactions [122,123,124,152]. 

At this stage, it would be fair to say that base metal catalysis is in its adolescence rather than infancy as many challenges remain unsolved. Despite the wide number of complexes available to choose from in catalytic hydrofunctionalization, a sufficiently broad scope of the substrate is not yet tolerated. Most published reports do not show good catalytic activity when substrates bearing hydroxy, carboxyl, and nitro functional groups are used. Typically, substrates containing halogens, electron-withdrawing, or electron-donating groups and esters dominate tables of substrate scope. Therefore, there are still opportunities to develop catalysts, which are more robust in the presence of less benign functionality.

Elucidation of reaction mechanisms allows us to better understand the most crucial factors in designing more efficient catalysts. However, the paramagnetic nature of many base metal complexes makes mechanistic studies difficult using traditional methods. Thus, the number of mechanistic studies at present remain limited; many proposed mechanisms are speculative at best and draw on the broad base of knowledge from precious metal catalysis. In this area, the application of computational methods has been warmly welcomed by the synthetic community. Of particular significance is the very recent work of Gade and co-workers who reported the first example of a metal-hydride peak for a paramagnetic species detected using ^1^H-NMR; a broad iron hydride resonance peak was detected at various temperatures [170]. Elegant mechanistic studies from Gade helped establish an experimental framework from which a carbonyl hydrosilylation mechanism was proposed. Moreover, recent advances by the Neidig group promised a renewed focus on the mechanistic study of iron-catalyzed reactions [171,172,173,174]. Exciting new opportunities still exist in the field of base metal catalysis, and one can expect exciting developments in the near future.
